# Supplementary material for: Content-rich biological network constructed by mining PubMed abstracts
Source: BMC Bioinformatics. 2004 Oct 8;5:147. doi: 10.1186/1471-2105-5-147 (PMC528731; doi:10.1186/1471-2105-5-147)
Supplement: Additional File 2 — The original results of the above study (non-essential files are deleted to keep the file size under the limit set by BMC bioinformatics). [file 1471-2105-5-147-S2.bz2 › chilibotAdditionalFile2/dip20/462ID10498628E10483/html/CCR9_CCL25.html]

 


 **CCR9** and **CCL25** 
  
Found 24 abstracts in PubMed, retrieved 20.  
 

 What does Google say? 
 PDF only 
| .edu only 

---

**Interactive relationship** (e.g. stimulation, inhibition, etc)

**Neutral relationship**- Role of the CC chemokine receptor 9  [ **CCR9** ]  TECK  [ **CCL25** ]  interaction in apoptosis.  Ref: 11997671 Apoptosis, 2002
- Our results suggest that TECK  [ **CCL25** ]  delivers signals through  **CCR9**  important for the navigation of developing thymocytes.  Ref: 10623805 J Immunol, 2000
- All  **CCR9**  thymocyte subsets migrated in response to  **CCL25** .  Ref: 11751956 J Immunol, 2002
- **CCR9**  mediates chemotaxis in response to  **CCL25**  thymus expressed chemokine  [ **CCL25** ]  and is selectively expressed on T cells in the thymus and small intestine.  Ref: 11884450 J Immunol, 2002
- Freshly isolated murine CD8alpha alpha CD3 IEL migrated in response to  **CCL25**  and expressed the  **CCL25**  receptor,  **CCR9** .  Ref: 12442331 Eur J Immunol, 2002
- **CCR9**  mediates chemotaxis of thymocytes in response to  **CCL25**  thymus expressed chemokine  [ **CCL25** ] , and its mRNA is selectively expressed in thymus and small intestine, the two known sites of T lymphopoiesis.  Ref: 11751956 J Immunol, 2002
- CC chemokine receptor CCR 9  [ **CCR9** ] , the receptor for the CC chemokine  **CCL25**  thymus expressed chemokine  [ **CCL25** ]  TECK  [ **CCL25** ] , is mainly expressed by thymocytes and by intraepithelial IEL and lamina propria lymphocytes of the small intestine.  Ref: 11675330 Blood, 2001
- Taken together, these results suggest that in thymocytes,  **CCR9**  is the only physiologic receptor for TECK  [ **CCL25** ]   **CCL25** ,.  Ref: 11675330 Blood, 2001
- Thymus expressed chemokine  [ **CCL25** ]  TECK  [ **CCL25** ]  or Ckbeta 15  **CCL25** , which signals through the chemokine receptor  **CCR9** , is constitutively expressed in the thymus and small intestine but not colon, and chemoattracts a small fraction of PBLs that coexpress the integrin alpha 4 beta 7.  Ref: 11046037 J Immunol, 2000
- TECK  [ **CCL25** ]  specifically induces a calcium flux and chemotaxis of GPR 9 6  [ **CCR9** ]  transfected cells.  Ref: 10623805 J Immunol, 2000
- Collectively, our data suggest that the selective expression of TECK  [ **CCL25** ]  in the small bowel underlie the homing of  **CCR9**  intestinal memory T cells to the small bowel rather than to the colon.  Ref: 11046037 J Immunol, 2000
- Consistent with this, CD8 lymphocytes from lymph nodes, spleen, and Peyer patches express a functional  **CCR9** , as its expression correlates with migration in response to  **CCL25** .  Ref: 11159507 Blood, 2001
- Characterization of  **CCR9**  expression and  **CCL25**  thymus expressed chemokine  [ **CCL25** ]  responsiveness during T cell development CD3 high CD69 thymocytes and gammadeltaTCR thymocytes preferentially respond to  **CCL25** .  Ref: 11751956 J Immunol, 2002
- Furthermore we show that TECK  [ **CCL25** ]  thymus expressed chemokine  [ **CCL25** ] , a chemokine produced by thymic medullary dendritic cells, is a functional ligand for GPR 9 6  [ **CCR9** ] .  Ref: 10623805 J Immunol, 2000
- AIMS Thymus expressed chemokine  [ **CCL25** ]  TECK  [ **CCL25** ]  or  **CCL25**  is selectively expressed in the small bowel SB, where lamina propria lymphocytes LPL and intraepithelial leukocyte expressing the cognate chemokine receptor  **CCR9**  predominate.  Ref: 11487533 Gastroenterology, 2001
- It is shown here that engagement of  **CCR9**  with TECK  [ **CCL25** ]  leads to phosphorylation of Akt, mitogen activated protein kinases, glycogen synthase kinase 3 beta, and a forkhead transcription factor, FKHR, in a human T cell line, MOLT4, that naturally expresses  **CCR9** .  Ref: 11493434 Blood, 2001

**Non-interactive relationship** (e.g. studied together, co-existance, homology, etc.)

- In contrast,  **CCR9** , whose ligand TECK  [ **CCL25** ]   **CCL25**  is predominantly restricted to the small intestine and thymus, is expressed by a fraction of IgA Ab secreting cells and almost all T cells in the small intestine, but by only a small percentage of plasma cells and plasmablasts in other sites.  Ref: 12671049 J Clin Invest, 2003
- Approximately one half of all gammadeltaTCR thymocytes and peripheral gammadeltaTCR T cells expressed  **CCR9**  on their surface, and these cells migrated in response to  **CCL25** .  Ref: 11751956 J Immunol, 2002
- CONCLUSIONS In SB immune mediated diseases, there is repartitioning of  **CCR9**  lymphocytes between SB and blood and an altered pattern of TECK  [ **CCL25** ]  expression in SB Crohn s.  Ref: 11487533 Gastroenterology, 2001
- Thus, oral antigens induce effector memory cells that express essential receptors for intestinal homing, namely the integrin alpha4beta7 and  **CCR9** , the receptor for the gut associated chemokine TECK  [ **CCL25** ]   **CCL25**  refs, 9.  Ref: 12840763 Nature, 2003
- These results imply a restricted role for lymphocyte  **CCR9**  and its ligand TECK  [ **CCL25** ]  in the small intestine, and provide the first evidence for distinctive mechanisms of lymphocyte recruitment that may permit functional specialization of immune responses in different segments of the gastrointestinal tract.  Ref: 10974041 J Exp Med, 2000
- The TECK  [ **CCL25** ]   **CCR9**  ligand receptor pair may play an important role in the pathogenesis of SB Crohn s disease.  Ref: 11487533 Gastroenterology, 2001
- We characterize the role of TECK  [ **CCL25** ]  and  **CCR9**  expresing lymphocytes in small intestinal Crohn s disease.  Ref: 11487533 Gastroenterology, 2001
- The role of thymus expressed chemokine  [ **CCL25** ]  and its receptor  **CCR9**  on lymphocytes in the regional specialization of the mucosal immune system.  Ref: 11046037 J Immunol, 2000
- When tested in chemotaxis assay, thymocytes isolated from  **CCR9**  mice failed to respond to TECK  [ **CCL25** ]   **CCL25** .  Ref: 11675330 Blood, 2001
- Analysis of  **CCR9**  expression in thymocytes from mice of different ages showed that  **CCR9**  levels are affected by age, as this receptor is more abundant, and its response to  **CCL25**  is more potent in newborn animals.  Ref: 11159507 Blood, 2001
- The TECK  [ **CCL25** ]  induced chemotaxis was sensitive to pertussis toxin and partially inhibited by Abs to  **CCR9** .  Ref: 11046037 J Immunol, 2000
- Murine  **CCR9** , a chemokine receptor for thymus expressed chemokine  [ **CCL25** ]  that is up regulated following pre TCR signaling.  Ref: 10623805 J Immunol, 2000
- Together these results demonstrate an important role for chemokines in the localization of T lymphocytes to the small intestinal mucosa and suggest that targeting  **CCL25**  and or  **CCR9**  may provide a means to selectively modulate small intestinal immune responses.  Ref: 12393847 J Clin Invest, 2002
- Thus, these results demonstrate that the  **CCL25**   **CCR9**  pathway is essential for CP formation and the consequent appearance of IEL.  Ref: 12096027 Int Immunol, 2002
- **CCR9** , the specific receptor for the beta chemokine TECK  [ **CCL25** ]   **CCL25** , is selectively expressed in thymus, lymph node, and spleen.  Ref: 11159507 Blood, 2001
- Pivotal role of  **CCL25**  TECK  [ **CCL25** ]   **CCR9**  in the formation of gut cryptopatches and consequent appearance of intestinal intraepithelial T lymphocytes.  Ref: 12096027 Int Immunol, 2002
- These recently activated  **CCR9**  CD8alphabeta lymphocytes selectively localized to the small intestinal mucosa, and in vivo neutralization of the  **CCR9**  ligand,  **CCL25** , reduced the ability of these cells to populate the small intestinal epithelium.  Ref: 12393847 J Clin Invest, 2002
- Adhesion was abolished in the presence of soluble ELC, SLC CCR7 ligands, and TECK  [ **CCL25** ]  a  **CCR9**  ligand.  Ref: 10706668 J Immunol, 2000
- **CCR9**  positive lymphocytes and thymus expressed chemokine  [ **CCL25** ]  distinguish small bowel from colonic Crohn s disease.  Ref: 11487533 Gastroenterology, 2001
- Lymphocyte CC chemokine receptor 9  [ **CCR9** ]  and epithelial thymus expressed chemokine  [ **CCL25** ]  TECK  [ **CCL25** ]  expression distinguish the small intestinal immune compartment Epithelial expression of tissue specific chemokines as an organizing principle in regional immunity.  Ref: 10974041 J Exp Med, 2000
